# High frequency of corticosteroid and immunosuppressive therapy in patients with systemic sclerosis despite limited evidence for efficacy

**DOI:** 10.1186/ar2634

**Published:** 2009-03-04

**Authors:** Nicolas Hunzelmann, Pia Moinzadeh, Ekkehard Genth, Thomas Krieg, Walter Lehmacher, Inga Melchers, Michael Meurer, Ulf Müller-Ladner, Thorsten M Olski, Christiane Pfeiffer, Gabriela Riemekasten, Eckhard Schulze-Lohoff, Cord Sunderkoetter, Manfred Weber, the German Network for Systemic Scleroderma Centers

**Affiliations:** 1 Department of Dermatology and Venerology, University of Cologne, Kerpener Straße 62, Cologne 50924, Germany; 2 Hospital of Rheumatology, Burtscheider Markt 24, Aachen 52066, Germany; 3 Institute of Medical Statistics, Informatics and Epidemiology, University of Cologne, Kerpener Straße 62, Cologne 50924, Germany; 4 Clinical Research Unit for Rheumatology, University Medical Center Freiburg, Hugstetter Straße 49, Freiburg 79106, Germany; 5 Department of Dermatology, Dresden University Hospital, Fetscherstraße 74, Dresden 01307, Germany; 6 Department of Rheumatology and Clinical Immunology, Kerckhoff Clinic, Benekestraße 2-8, Bad Nauheim 61231, Germany; 7 Rheumatology and Clinical Immunology, Charité, Charitéplatz 1, Berlin 10117, Germany; 8 Medical Clinic I, Hospital Cologne-Merheim, Ostmerheimer Straße 200, Cologne 51109, Germany; 9 Department of Dermatology, University of Münster, Von-Esmarch-Straße 58, Münster 48149, Germany

## Abstract

**Introduction:**

In systemic sclerosis (SSc) little evidence for the effectiveness of anti-inflammatory and immunosuppressive therapy exists. The objective of this study was to determine the extent to which SSc patients are treated with corticosteroids and immunosuppressive agents.

**Methods:**

Data on duration and dosage of corticosteroids and on the type of immunosuppressive agent were analyzed from 1,729 patients who were registered in the German Network for Systemic Scleroderma (DNSS).

**Results:**

A total 41.3% of all registered SSc patients was treated with corticosteroids. Corticosteroid use was reported in 49.1% of patients with diffuse cutaneous SSc and 31.3% of patients with limited cutaneous SSc (*P *< 0.0001). Among patients with overlap disease characteristics, 63.5% received corticosteroids (*P *< 0.0001 vs. limited cutaneous SSc). A total 16.1% of the patients received corticosteroids with a daily dose ≥ 15 mg prednisone equivalent. Immunosuppressive therapy was prescribed in 35.8% of patients. Again, among those patients with overlap symptoms, a much higher proportion (64.1%) was treated with immunosuppressive agents, compared with 46.4% of those with diffuse cutaneous SSc sclerosis and 22.2% of those with limited cutaneous SSc (*P *< 0.0001). The most commonly prescribed drugs were methotrexate (30.5%), cyclophosphamide (22.2%), azathioprine (21.8%) and (hydroxy)chloroquine (7.2%). The use of these compounds varied significantly between medical subspecialties.

**Conclusions:**

Despite limited evidence for the effectiveness of corticosteroids and immunosuppressive agents in SSc, these potentially harmful drugs are frequently prescribed to patients with all forms of SSc. Therefore, this study indicates the need to develop and communicate adequate treatment recommendations.

## Introduction

Systemic sclerosis (SSc) is a rare autoimmune disease involving the skin and internal organs. The disease hallmark is an overproduction and accumulation of collagen and other extracellular matrix proteins, resulting in thickening of the skin and fibrosis of the affected organs (for example, gastrointestinal tract, lung, heart, and kidney). The etiology of SSc is still not fully elucidated, but the dominant phenomena are immunologic mechanisms, vascular endothelial cell injury and an activation of fibroblasts.

Significant advances have been made during recent years in symptomatic organ-specific therapy [1]. Only few controlled clinical studies, however, have been performed for drugs with anti-inflammatory, immunosuppressive or anti-fibrotic properties. The rarity of SSc, several disease subsets and a highly variable course of disease are major obstacles to performing adequately designed studies with a sufficient number of patients [2].

Corticosteroids are still the mainstay of treatment for most autoimmune diseases. There is no randomized controlled study addressing the use of corticosteroids in SSc, however, that demonstrates improvement of skin fibrosis or organ involvement [3]. In contrast, it has been observed that high-dose corticosteroid application (≥ 15 mg/day) may contribute to the development of renal crisis [4].

Similarly, large well-controlled prospective clinical trials are lacking for most of the drugs with immunosuppressive or anti-fibrotic properties that have been used in the treatment of SSc. The small number of controlled studies that were performed failed to demonstrate a significant benefit – with the exception of cyclophosphamide, which showed a modest, statistically significant benefit in a recent randomized controlled trial [5], and to a limited extent methotrexate for early active disease [6].

The lack of data on the frequency and extent to which SSc patients are treated with corticosteroids or immunosuppressive agents in clinical practice, few well-controlled clinical studies and the limited effectiveness of available therapies are probably the main obstacles for the development of clinical recommendations or guidelines for immunosuppressive therapy of SSc patients.

In October 2003, the German Network for Systemic Scleroderma (Deutsches Netzwerk für Systemische Sklerodermie (DNSS)) was established to improve basic and clinical research for this complex disease. The network, which is funded by the German Federal Ministry of Education and Research, consists of rheumatologists, dermatologists, pulmonologists, and nephrologists, who established a nationwide patient registry to collect data from a sufficiently high number of patients to analyze diagnostic and therapeutic procedures.

For the present study, the registry of the network was used to investigate the prescription of corticosteroid and immunosuppressive therapy in a large number of SSc patients. The study demonstrates the widespread use of glucocorticoids and the large variety of immunosuppressive agents used, despite the lack of evidence for their effectiveness. This result reflects also characteristic problems associated with the treatment of rare, chronic autoimmune diseases in general. Both the physician and the patient are confronted with a severe, potentially life-threatening and difficult to treat chronic disease. Both parties, to varying degrees, therefore feel obliged to intervene therapeutically, despite the lack of well-performed clinical trials and corresponding therapeutic guidelines.

## Materials and methods

The DNSS presently consists of 27 clinical centers embracing different subspecialties; that is, rheumatology (11 centers), dermatology (13 centers), pulmonology (two centers), and nephrology (one center), the latter being nationally known for their expertise in complications of pulmonary or renal involvement of SSc. The patient population reflects both those cared for by centers of expertise in scleroderma and private practitioners in a variety of specialties.

The study, including an informed consent regarding data storage, has been approved by the lead ethics committee of the Cologne University Hospital and by the respective ethics committee of the centers.

By August 2007 more than 1,729 patients had been registered in the database. Patients with the diagnosis of undifferentiated scleroderma and SSc sine scleroderma were excluded from this analysis on corticosteroid and immunosuppressive therapy to enable comparison with other independent populations. The analysis thus focused on a total of 1,416 registered patients, grouped into diffuse cutaneous SSc (34.7%; 492/1,416 patients), limited cutaneous SSc (53.4%; 756/1,416 patients) and overlap subsets (11.9%; 168/1,416 patients) (Table 1).

**Table 1:** Patient characteristics

	Total	Missing data	Limited cutaneous systemic sclerosis	Diffuse cutaneous systemic sclerosis	Overlap syndrome
Characteristic					
Number of patients (n (%))	1,419 (100%)	0.9	56 (53.4%)	492 (34.7%)	168 (11.9%)
Female	82.8	0.1	88.0	74.9	85.1
Male	17.2	0.1	12.0	25.1	14.9
Antinuclear antibodies (ANA)-positive	91.5	0.5	89.9	91.9	95.8
Scl 70-positive	30.9	0.7	18.5	55.6	11.4
Anticentromere antibodies (ACA)-positive	36.6	2.5	59.7	10.2	16.2
Organ involvement by systemic sclerosis subset					
Raynaud phenomenon	95.8	0.2	96.3	95.1	96.4
Skin involvement	93.5	0.4	92.8	97.5	83.9
Pulmonary hypertension	14.2	0.4	12.8	17.7	9.5
Pulmonary fibrosis	38.0	0.4	24.6	59.1	33.3
Esophagus	63.6	0.3	61.1	67.4	64.9
Stomach	15.2	0.5	15.8	14.5	14.9
Intestine	5.7	0.5	6.3	5.1	5.4
Kidney	12.3	0.5	10.3	16.1	7.1
Heart	14.9	0.5	11.2	21.2	11.3
Musculoskeletal system	51.0	8.1	45.1	53.7	68.3
Nervous system	6.2	1.8	5.7	5.6	10.1
Sicca symptoms	42.0	2.3	45.5	36.9	43.4
Masticatory organ	30.3	12.4	25.3	38.6	27.5

Data presented as percentages unless stated otherwise.

Complete data sets on corticosteroid therapy were available for 1,396 registered patients and data sets on immunosuppressive therapy for 1,394 registered patients with the diffuse cutaneous SSc, limited cutaneous SSc and overlap subsets.

The data, which are entered in the patient registry, are collected on a four-page patient registration form – which was adopted by the Network via consensus, and which is used by all contributing clinical centers. The patient registration form records data on gender, date of birth, disease subsets as well as information on organ involvement, antibodies or current symptoms as published previously [7]. A separate page solicits information on drug and other therapies. Data on corticosteroid and immunosuppressive therapy are given as milligrams per day. Two reference documents (that is, a set of definitions for the items on the registration form, and recommendations for organ-specific diagnostic procedures) were prepared to ensure consistency of registered patients' data in the network centers.

Organ involvement was defined as recently described in detail [7]. To ensure the detection of disease heterogeneity, the registry defined five subsets – limited cutaneous SSc [8], diffuse cutaneous SSc [8], overlap syndrome [9,10], SSc sine scleroderma [11-13], and undifferentiated connective tissue disease with features of scleroderma [14,15] – as recently described [7]. The overlap subset was defined as SSc according to American College of Rheumatology criteria or the main symptoms of SSc, combined with another nonorgan-specific autoimmune disease or with the main symptoms of such a disease and, as a rule, with proof of characteristic autoantibodies (for example, anti-U1-RNP or anti-PMScl).

### Data recording and statistical analyses

The DNSS maintains a centralized online patient registry in which all patient data from the four-page DNSS-patient registration form are entered. A Central Office for Coordination, set up at the Department of Dermatology and Venerology at the University of Cologne, acts as the data manager. The DNSS closely cooperates with the Center for Clinical Studies Cologne (ZKS Koeln), which, on the basis of the registration form, designed the online patient registry using the MACRO software for Clinical Trials. Seven clinical centers currently use the option to register their patients online. The remaining centers perform their registration on paper and send the filled-in questionnaires to the Central Office for Coordination, where the registration forms are validated and entered into the online registry.

The data were statistically analyzed using Excel and SPSS 14.0 for tabular and graphic representation. Statistical evaluation was performed using contingency table tests (chi-square test or Fisher's exact *t *test) to describe significant differences or associations. In general, *P *values < 0.05 are mentioned. When multiple testes were performed, however, only values below 0.0001 were considered significant. For most variables, less than 5% of data were missing. In some sets, however, the percentage of missing data is higher; for example, the musculoskeletal system (synovitis and/or myositis based on clinical evaluation and raised serum muscle enzyme levels, joint contracture and muscle weakness), masticatory organ (microstomia, obvious decreased mouth opening, fibrosis of the lingual frenulum) and palpitations (subjective sensation of an increased, accelerated or irregular heartbeat). This is due to the fact that some parameters were added to the registration form after the registry had been initiated. Patients with missing data for the respective analysis were excluded from the analysis.

Furthermore, multivariate logistic analyses with particular attention to multiple collinearity were performed to assess which factors might be associated with the use of corticosteroid or immunosuppressive agents. Odds ratios and the corresponding 95% confidence intervals were calculated.

The evaluation focused on a comparison between the use of different corticosteroid dosages and immunosuppressive agents related to the following variables: SSc subset (diffuse cutaneous SSc, limited cutaneous SSc, overlap syndrome), antibody status (Scl-70, centromere), organ involvement (gastrointestinal tract, lung, heart, kidney, musculoskeletal system, skin) as well as laboratory/clinical parameters (for example, erythrocyte sedimentation rate (ESR), proteinuria, modified Rodnan skin score) and medical subspecialty (that is, rheumatologists, dermatologists).

## Results

### Corticosteroid use

**Figure 1:**
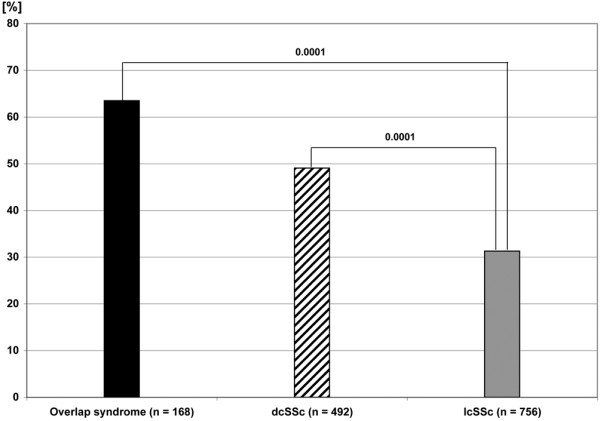
Use of corticosteroids in systemic sclerosis subtypes. dcSSc, diffuse cutaneous systemic sclerosis; lcSSc, limited cutaneous systemic sclerosis.

Complete data sets on corticosteroid therapy were available for 1,396 out of 1,416 patients. A total 41.3% of SSc patients (577/1,396) received corticosteroids; 73.3% of these patients received additional immunosuppressive therapy. The use of corticosteroid therapy varied considerably in the different disease subsets, with overlap syndrome displaying the highest frequency (63.5%) (see Figure 1). Internal organ involvement was associated with varying degrees of corticosteroid use. Lung fibrosis was associated with the highest frequency (55.6%), followed by renal involvement (56.5%), cardiac involvement (51.2%), musculoskeletal involvement (47.9%), pulmonary hypertension (44.7%) and gastrointestinal involvement (43.0%).

Male patients received corticosteroids more often than female patients (52.5% (127/242) vs. 38.9% (449/1,153)) (*P *< 0.0001). An ESR above 30 mm/hour resulted in more frequent corticosteroid use (48.3%), compared with values below 30 mm/hour (40.2%) (*P *< 0.02). Patients with cutaneous involvement alone (n = 447) received corticosteroids (27.1%) and/or immunosuppressive agents (21.5%) to a lesser degree compared with patients suffering from lung and musculoskeletal manifestations (121/447).

Exact dosages of corticosteroid therapy were documented for 466 patients, and revealed that 16.9% (79/466) of patients were given dosages of prednisone equivalents ≥ 15 mg/day, 28.9% (135/466) received dosages <15 mg/day and ≥ 7.5 mg/day, and 54.1% of patients (252/466) received dosages below 7.5 mg/day. Patients with an elevated ESR constituted a higher proportion of patients were treated with dosages >7.5 mg/day (32.5% vs. 20.2%) (*P *< 0.005). Dosages <15 mg/day were significantly associated with the limited cutaneous SSc subset (*P *< 0.009), whereas dosages ≥ 15 mg/day were associated with the overlap syndrome (*P *< 0.03).

Since corticosteroids have been described as a risk factor for renal involvement and for renal crisis, we analyzed the frequency of corticosteroid use in patients with proteinuria (42.9%; 60/140), hypertension (44.1%; 138/313) or renal insufficiency (47.7%; 95/199). In the majority of patients, renal insufficiency was not due to renal crisis (unpublished observation). Multivariate analysis (Table 2) showed that kidney involvement was indeed associated with corticosteroid use. During the observation period, however, there was no statistically significant difference in kidney involvement between the two different corticosteroid dosage groups (<15 mg vs. ≥ 15 mg).

**Table 2:** Multivariate relationship of corticosteroid and immunosuppressive agent use with subspecialty, disease subset and organ involvement

Variable	Corticosteroid therapy vs. no corticosteroid therapy			Immunosuppressive therapy vs. no immunosuppressive therapy		
		
	Odds ratio	95% confidence interval	*P *value	Odds ratio	95% confidence interval	*P *value
Subspecialties (rheumatology vs. dermatology)	1.54	1.19 to 1.98	0.001	2.46	1.88 to 3.21	<0.0001
Gender (female vs. male)	0.69	0.51 to 0.96	0.025	0.78	0.56 to 1.10	0.13
Age (years)	0.99	0.99 to 1.01	0.67	0.99	0.98 to 1.00	0.04
Systemic sclerosis subsets						
Limited cutaneous systemic sclerosis (reference group)	1.00			1.00		
Diffuse cutaneous systemic sclerosis	1.47	0.45 to 4.88	0.53	1.77	0.51 to 6.19	0.37
Overlap syndrome	3.64	1.06 to 12.51	0.04	3.69	1.02 to 13.37	0.05
Organ involvement						
Pulmonary hypertension	0.83	0.59 to 1.17	0.30	0.94	0.66 to 1.35	0.73
Pulmonary fibrosis	2.30	1.77 to 2.99	<0.0001	1.53	1.16 to 2.00	0.002
Musculoskeletal system	1.40	1.08 to 1.81	0.01	1.31	1.00 to 1.71	0.05
Esophagus	1.01	0.78 to 1.29	0.96	0.92	0.70 to 1.20	0.53
Kidney	1.66	1.12 to 2.47	0.01	0.91	0.59 to 1.38	0.64
Heart	1.28	0.91 to 1.81	0.16	1.34	0.93 to 1.92	0.12

Corticosteroids were prescribed significantly more often by rheumatologists (48.3%; 360/746) than by dermatologists (33.4%; 217/650) (*P *< 0.0001) (Table 3). This difference cannot be ascribed to disease severity since no significant differences were found in organ involvement in the two patient groups other than musculoskeletal involvement or sicca symptoms. Multivariate logistic analysis accounting for differences in subset frequency and organ involvement confirmed this observation (Table 2).

**Table 3:** Differences between corticosteroid and immunosuppressive therapy use between rheumatological and dermatological centers of the DNSS

Treatment	Rheumatological centers (%)	Dermatological centers (%)	*P *value
Corticosteroids	48.3 (360/746)	33.4 (217/650)	<0.0001
Immunosuppressants	45.6 (340/745)	24.5 (159/649)	<0.0001
Cyclophosphamide	21.5 (73/340)	23.9 (38/159)	
Azathioprine	21.8 (74/340)	22.0 (35/159)	
Methotrexate	35.9 (122/340)	18.9 (30/159)	<0.0001
(Hydroxy)chloroquine	8.8 (30/340)	3.8 (6/159)	<0.04

DNSS, Deutsches Netzwerk für Systemische Sklerodermie (German Network for Systemic Scleroderma).

### Immunosuppressive agents

**Figure 2:**
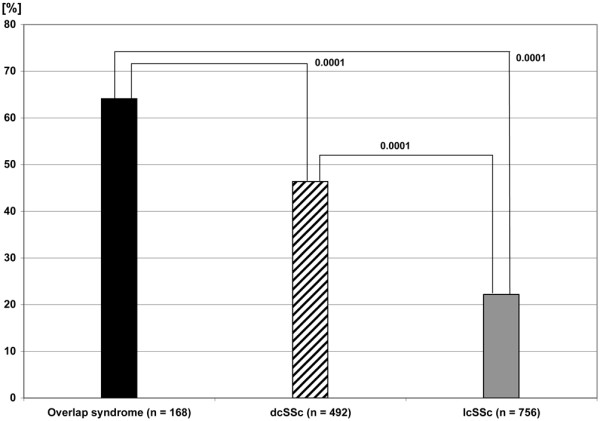
Use of immunosuppressive agents in systemic sclerosis subtypes. dcSSc, diffuse cutaneous systemic sclerosis; lcSSc, limited cutaneous systemic sclerosis.

In the study population, 35.8% (499/1,394) of patients were treated with immunosuppressive agents. Patients suffering from the diffuse type of SSc received immunosuppressive drugs more often (46.4%; 225/485) than individuals with limited cutaneous SSc (22.2%; 165/742) (*P *< 0.0001) (see Figure 2).

Male patients (46.9%; 113/241) were more frequently treated with these drugs than female patients (33.4%; 385/1,152) (*P *< 0.0001). The frequency of organ involvement associated with the use of immunosuppressive agents varied considerably. Lung fibrosis was associated with the highest frequency (44.4%; 236/531), followed by cardiac involvement (44.7%; 92/206), musculoskeletal involvement (43.4%; 285/657), pulmonary hypertension (40.1%; 79/197), renal involvement (38.7%; 65/168) and gastrointestinal involvement (36.7%; 327/891). Elevation of the ESR above 30 mm/hour was also associated with an increased use of these agents (44.6% (119/267) vs. 34.9% (337/965)) (*P *< 0.004). Patients with cutaneous involvement alone (n = 447) received corticosteroids (27.1%) and/or immunosuppressive agents (21.5%) to a lesser degree compared with patients suffering from lung and musculoskeletal manifestations.

**Figure 3:**
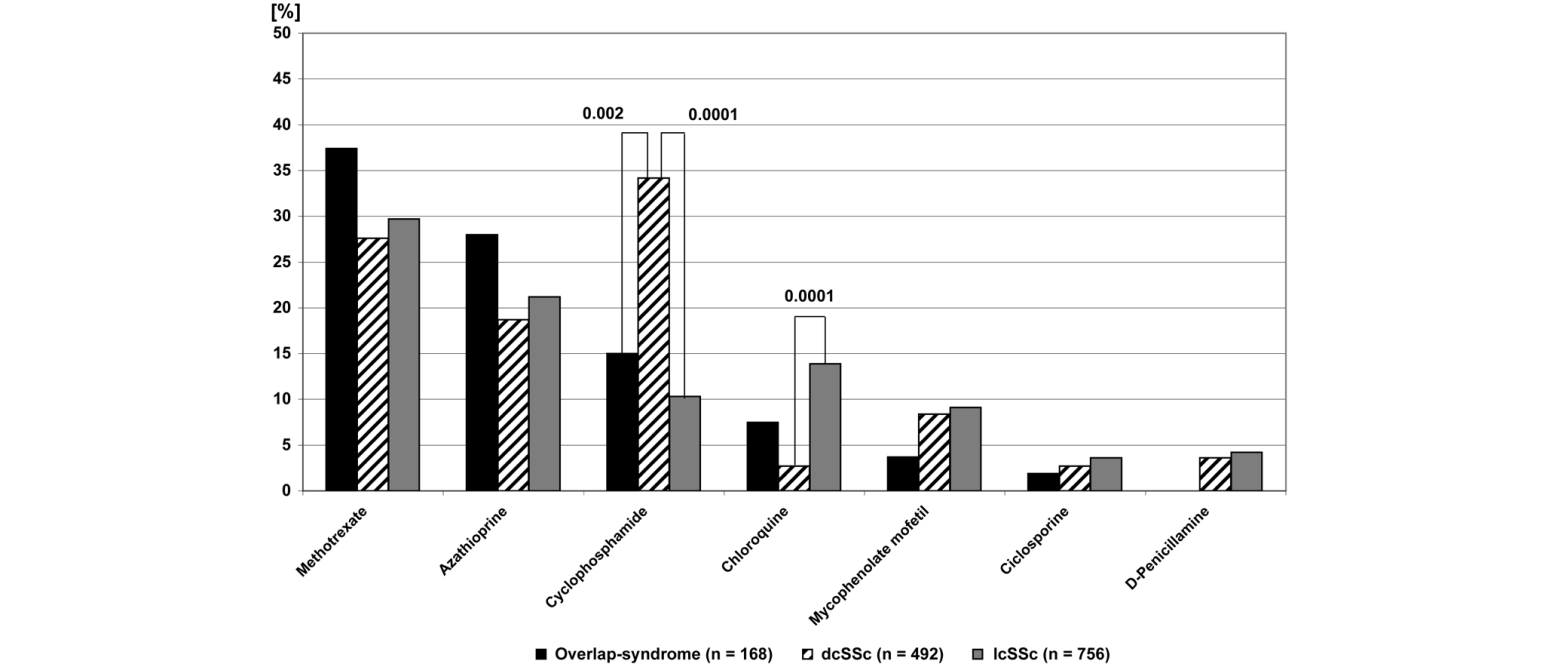
Use of different immunosuppressive agents in systemic sclerosis subtypes. dcSSc, diffuse cutaneous systemic sclerosis; lcSSc, limited cutaneous systemic sclerosis.

The immunosuppressive agents used, in order of decreasing frequency, are methotrexate (30.5%; 152/499), cyclophosphamide (22.2%; 111/499), azathioprine (21.8%; 109/499), (hydroxy)chloroquine (7.2%; 36/499) and mycophenolate mofetil (7.6%; 38/499). Cyclosporine A (3%; 15/499) and D-penicillamine (3.2%; 16/499) were prescribed only on rare occasions (Table 4). The use of the different immunosuppressive agents in disease subsets is shown in Figure 3. Combination therapy with corticosteroids was frequent on average in 65.7% of patients, with no significant difference in frequency between the various immunosuppressive agents used (Table 4).

**Table 4:** Frequency of use of immunosuppressive agents and combination with corticosteroids

	Immunosuppressive agent		Combination with corticosteroids	
		
	Total number	%	Total number	%
Methotrexate	(152/499)	30.5	(101/152)	66.4
Cyclophosphamide	(111/499)	22.2	(81/111)	73.0
Azathioprine	(109/499)	21.8	(75/109)	68.8
(Hydroxy)chloroquine	(36/499)	7.2	(20/36)	55.6
Mycophenolate mofetil	(38/499)	7.6	(26/38)	68.4
Cyclosporine	(15/499)	3.0	(9/15)	60.0
D-Penicillamine	(16/499)	3.2	(7/16)	43.8

The relative frequency of the immunosuppressive agents used in the different subsets is shown in Figure 3. In the diffuse subset the probability of the use of cyclophosphamide was significantly higher and the use of (hydroxy)chloroquine significantly lower when compared with other immunosuppressants, whereas in the limited subset the probability to be treated with cyclophosphamide was significantly reduced.

No alterations in prescription frequencies for immunosuppressive agents were found when the first 850 patients were compared with patients 850 to 1,700 (data not shown). Significant differences were noted, however, when prescription frequencies were compared between rheumatologists and dermatologists for the use of methotrexate, mycophenolate mofetil and, to a lesser degree, for (hydroxy)chloroquine (Table 3).

## Discussion

For most rare diseases there is a lack of therapeutic recommendations or guidelines, owing to the poor database, the difficulty to perform clinical studies with sufficient statistical power and an often marked clinical heterogeneity in these patients.

SSc was therefore chosen as a paradigm to investigate the use of corticosteroid therapy and immunosuppressive therapy in a rare, severe disease with limited evidence for its efficacy and an absence of validated recommendations or guidelines.

To this end, treatment was analyzed in patients whose data were recorded in the registry of the DNSS. The patient population comprises patients of the clinical centers specializing in the care of SSc patients participating in the network, but also of patients who are seen only once a year in a clinical center participating in the network and otherwise receive care from a rheumatologist, a dermatologist or a general practitioner in private practice.

Corticosteroids are still a mainstay of treatment in most autoimmune rheumatic conditions. The use of corticosteroids in SSc is controversial as steroids inhibit proteases that increase connective tissue turnover, thus counteracting fibrosis [16]. More importantly, high-dose prednisone therapy has been associated in a retrospective case–control study with an increased risk of developing renal crisis [4,17], a severe complication of kidney involvement. No well-controlled study on the use of corticosteroids in SSc has been published to date. Nevertheless, experts share the opinion that glucocorticoids may be indicated in patients with inflammatory myositis, pericarditis, or alveolitis [1].

Our cross-sectional study reveals that SSc patients who suffer from diffuse skin affection or from overlap syndrome are treated more frequently with corticosteroids than patients with other SSc subsets. Corticosteroid use was not restricted to patients with clear signs of inflammation, however – such as, for example, elevated ESR, myositis, or synovitis.

Notably, a significant proportion of those individuals who received corticosteroids were already affected by renal dysfunction. About one-sixth of the patients who were treated with corticosteroids received prednisone doses ≥ 7.5 mg/day. This is an unexpected result, as higher doses of glucocorticoids have been suggested to play a role in facilitating acute renal failure [4,17]. The frequency of renal crisis was not assessed in the DNSS registry, and therefore no comparative data on patients affected by renal crisis can be provided. No significant difference in kidney involvement (that is, renal insufficiency, proteinuria) between the different corticosteroid dosage groups was evident in our study, however, which may be due to the relatively low number of patients in the high-dose group.

Having evaluated the overall use of immunosuppressive agents, we analyzed the frequency with which different compounds were used. The most commonly prescribed immunosuppressive drug in this study, especially for patients with overlap syndrome, was methotrexate. Methotrexate is a standard disease-modifying drug in rheumatoid arthritis, but so far results on its use in the treatment of SSc are still contradictory. The initially promising results with ameliorated skin score and ESR in early diffuse disease [6] were not confirmed by a follow-up study, in which neither the treatment group nor the control group experienced significant improvement during 1 year of treatment [18]. The high frequency of methotrexate prescription could reflect an increased use in patients with overlap syndrome presenting with signs of myositis or arthritis. Multivariate analysis accounting for these factors, however, failed to demonstrate a significantly increased use in these patients.

Azathioprine, originally introduced in transplantation medicine in the 1960s, is still widely used as corticosteroid-sparing agent in autoimmune diseases such as systemic lupus erythematosus, multiple sclerosis or bullous diseases of the skin. A randomized placebo-controlled clinical trial on axathioprine use in SSc is still lacking, however, while a recent uncontrolled study described its inferiority to cyclophosphamide [19]. Although there are no data supporting the use of azathioprine, the drug was therefore second in our study among the immunosuppressive agents prescribed.

Cyclophosphamide is frequently used as immunosuppressive drug in rheumatic diseases, such as Wegener's disease or systemic lupus erythematosus. With an oral daily dose between 1 and 2.5 mg/kg/day and together with prednisone, cyclophosphamide was able to improve pulmonary function and to increase survival in fibrosing alveolitis [3,20]. A placebo-controlled, randomized clinical trial recently confirmed these studies [5]. Despite such proof for a disease-modifying effect, cyclophosphamide is only ranked third in the frequency of immunosuppressive agents used. It was, however, the significantly most frequently used drug in the subset associated with the most severe disease course (that is, diffuse cutaneous SSc).

No studies are available on the effectiveness of (hydroxy)chloroquine – a drug largely used for skin involvement in lupus erythematosus or in mild cases of rheumatoid arthritis – in SSc. The reasons for use of cyclophosphamide and the preferred use in limited cutaneous SSc can therefore only be speculated, as no increased association was found for the presence of synovitis (data not shown) or in patients with overlap syndrome.

Mycophenolate mofetil, increasingly applied for the treatment of allograft rejection and kidney involvement in systemic lupus erythematosus, is used in a considerable number of patients in our study, preferably by dermatologists, despite the poor data on its effectiveness [21].

In uncontrolled studies, D-penicillamine was initially observed to have a positive effect on skin thickness, which was not confirmed by a double-blind randomized trial [22]. This lack of effectiveness and the considerable side effects of D-penicillamine presumably have led to its low frequency of use.

The high rate of side effects caused by cyclosporine, which, in addition, has repeatedly been reported to induce renal crisis in SSc patients [23], can also explain the low frequency of cyclosporine use we found in our study.

To identify possible differences in prescription habits between medical subspecialties, we stratified our data and compared prescription preferences in rheumatological and dermatological centers. Rheumatologists care more frequently for patients with overlap syndrome (*P *< 0.01, data not shown), which is usually characterized by prominent musculoskeletal involvement. Multivariate analysis, however, revealed that rheumatologists have a significantly higher preference for the use of corticosteroids and immunosuppressive agents in SSc patients, even after correction for the disease subset, musculoskeletal involvement or lung fibrosis as instances associated with increased inflammatory activity and the necessity for immunosuppressive treatment. In the absence of therapeutic guidelines, we assume that the different medical subspecialties are guided by current daily practice, relevant knowledge and therapeutic experience (that is, the use of methotrexate, azathioprine, (hydroxy)chloroquine) with disease-modifying therapy in other rheumatological diseases when deciding on an anti-inflammatory therapy in a severe autoimmune disease such as SSc.

Our findings are consistent with those of Pope and colleagues [24], who surveyed the therapeutic practice and the use of immunosuppressive agents and corticosteroids by members of the SSc clinical trials consortium, the Canadian Rheumatology Association and the American College of Rheumatology, with the exception of higher rates of D-penicillamine use in SSc in North America (that is, 20% for skin involvement).

## Conclusion

The present study is the first investigating the use of corticosteroids and immunosuppressive therapy in a large, well-defined cohort of SSc patients. The study reveals a widespread use of anti-inflammatory and immunosuppressive therapy of SSc patients in clinical practice and a marked therapeutic difference between disease subsets. The frequency with which these drugs are prescribed is in contrast to the weak data supporting their use, or which even argue against their use, as it is the case for higher doses of corticosteroids. Our analyses also suggest that, presumably due to the lack of treatment recommendations, specialists who care for SSc patients are guided by current treatment practices for other autoimmune diseases when deciding for or against a therapeutic option. The present study therefore underlines the urgent need to develop treatment recommendations for SSc. Specifically, these recommendations need to address the use of corticosteroids and immunosuppressive agents with respect to disease subsets, organ involvement and disease activity.

## Abbreviations

DNSS: Deutsches Netzwerk für Systemische Sklerodermie (German Network for Systemic Scleroderma); ESR: erythrocyte sedimentation rate; SSc: systemic sclerosis.

## Competing interests

The authors declare that they have no competing interests.

## Authors' contributions

NH and PM have made substantial contributions to conception of this manuscript, as well as to the analysis and interpretation of the data. They have made substantial contributions to patient recruitment and registration of patient data. These authors contributed equally to this work. TK has made substantial contributions to conception of this manuscript, as well as the analysis and interpretation of the data. He has given final approval of the version to be published. WL has made substantial contributions to statistical evaluation of the collected data. EG, IM, MM, UM-L, TMO, CP, GR, ES-L, CS, MW and the DNSS Centers have made substantial contributions to patient recruitment and registration of patient data. They are members of the expert centers of the DNSS. They contributed to writing this manuscript and have given final approval of the version to be published. Co-authors have also made substantial contributions to patient recruitment and registration of patient data. They are members of the DNSS. All authors read and approved the final manuscript.

## Authors' information

Co-authors: Michael Buslau (Reha Rheinfelden, Rheinfelden, Schweiz), Ina Kötter and Gerhard Fierlbeck (Interdisciplinary Centre for Immunology, Rheumatology and Autoimmune Diseases (INDIRA), University Hospital Tübingen, Germany), Frank Reichenberger (Department of Internal Medicine, University of Giessen, Germany), Adelheid Maria Müller and Rotraud Meyringer (Department of Internal Medicine, University of Regensburg, Germany), Margitta Worm and Pascal Klaus (Department of Dermatology, Venerology and Allergology, University Hospital Charité, Berlin, Germany), Kerstin Steinbrink (Department of Dermatology, University of Mainz, Germany), Annegret Kuhn (Department of Dermatology, University of Münster, Germany), Merle Haust (Department of Dermatology, University of Düsseldorf, Germany), Rüdiger Hein (Department of Dermatology and Allergology, University of Munich, Germany), Kurt Gräfenstein and Aaron Juche (Johanniter Hospital in Fläming gGmbH, Center of Rheumatology of Brandenburg, Treuenbrietzen, Germany), Hans-Martin Lorenz and Norbert Blank (Department of Internal Medicine, University of Heidelberg, Germany), Ralf Hinrichs (Hautarztpraxis am Ring, Cologne, Germany), Konrad Walker and Karin Scharffetter-Kochanek (Department of Dermatology and Allergology, University of Ulm, Germany), Elisabeth Aberer and Gabor Bali (Department of Dermatology, University of Graz, Austria), Enno Schmidt (Department of Dermatology, Allergology und Venerology, University Hospital Schleswig-Holstein/Campus Lübeck, Germany), Christoph Fiehn (Department of Internal Medicine, Center of Rheumatology, Baden-Baden, Germany), Ludwig Gross (Clinic for Rheumatology, Bad Bramstedt, Germany), Percy Lehmann (Department of Dermatology, Allergology and Environmental Medicine, Private University Witten-Herdecke, HELIOS Klinikum Wuppertal, Germany), Rudolf Stadler and Verena Bartels (Department of Dermatology, Clinic of Minden, Germany), Rolf-Markus Szeimies and Sigrid Karrer (Department of Dermatology, University of Regensburg, Germany), Cornelia Seitz (Department of Dermatology and Venerology, Georg-August-University of Göttingen, Germany), Kristian Reich (Dermatologikum Hamburg, Hamburg, Germany), Ivan Foeldvári (Hospital Eilbek, Hamburg, Germany), Andrea Rubbert (Department of Rheumatology, University of Cologne, Germany), Markus Böhm (Department of Dermatology, University of Münster, Germany), and Petra Saar (Department of Rheumatology and Clinical Immunology, Kerckhoff Clinic, Bad Nauheim, Germany).
